# Supplementary material for: The Forgotten Agenda of Wasting in Southeast Asia: Burden, Determinants and Overlap with Stunting: A Review of Nationally Representative Cross-Sectional Demographic and Health Surveys in Six Countries
Source: Nutrients. 2020 Feb 20;12(2):559. doi: 10.3390/nu12020559 (PMC7071426; doi:10.3390/nu12020559)
Supplement: Supplementary file 1 [file nutrients-12-00559-s001.pdf]

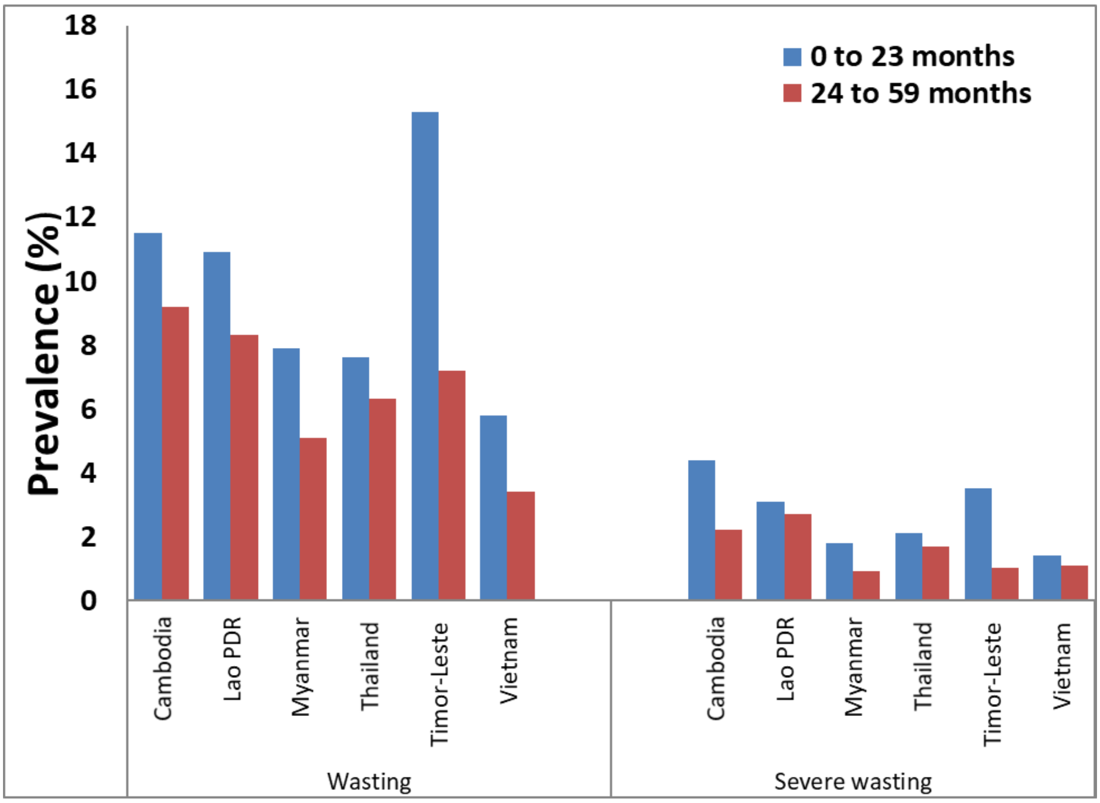

Figure S1: Prevalence of wasting and severe wasting among children 0 to 23months and 24 to 59 months.

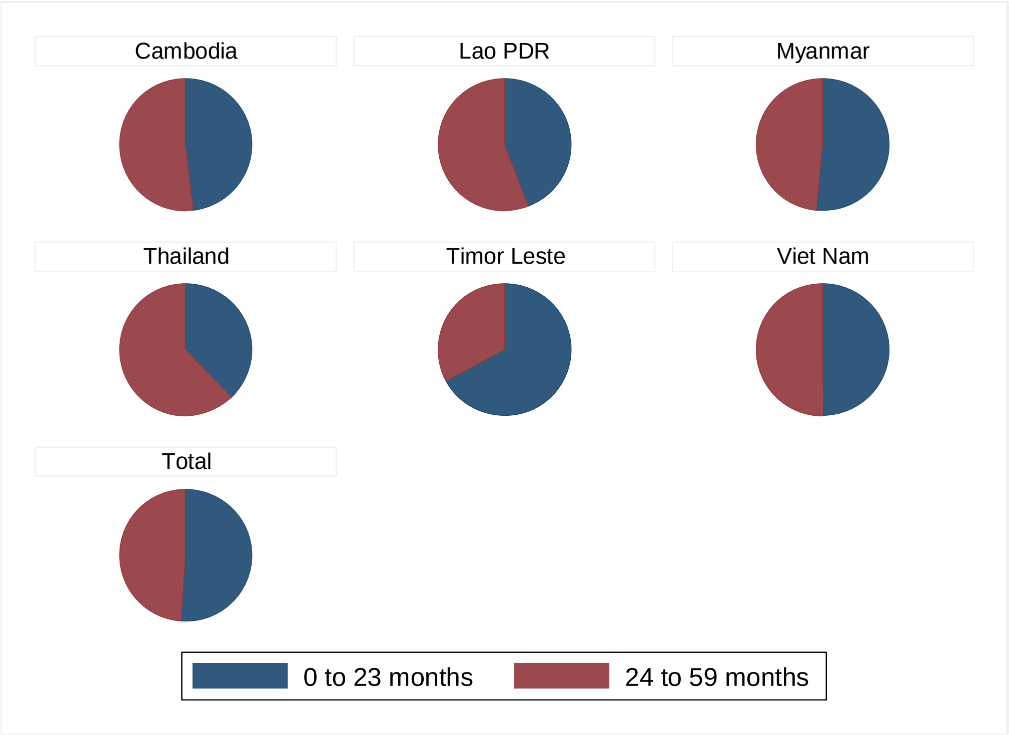

Figure S2. Contribution of the 0 to23 months and the 24 to 59 months age groups to wasting caseload.

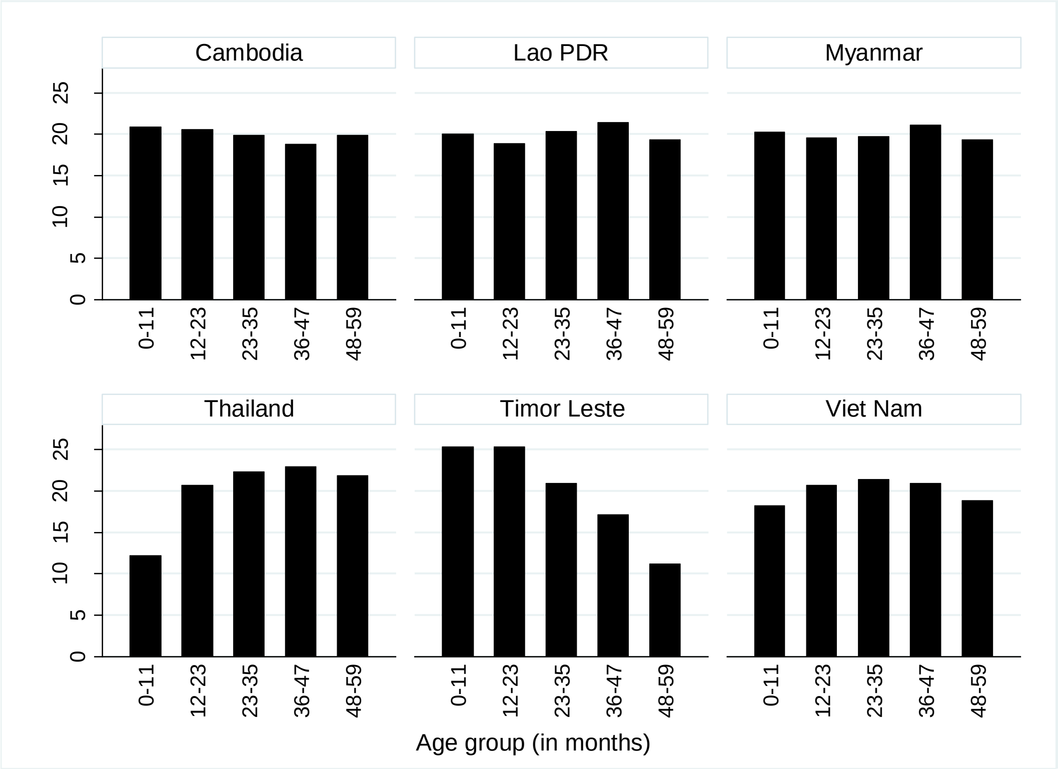

Figure S3. Distribution of the burden of wasting and concurrence across age group 12-months interval by country.
